# Summary of best evidence for prevention of postoperative pulmonary complications after surgery for patients undergoing gastric cancer operations

**DOI:** 10.3389/fonc.2025.1515502

**Published:** 2025-03-05

**Authors:** Mengnan Li, Guang Fu, Wenjuan Mo, Yuanyuan Yan

**Affiliations:** ^1^ School of Nursing, Hengyang Medical School, University of South China, Hengyang, Hunan, China; ^2^ Department of Gastrointestinal Surgery, The First Affiliated Hospital of University of South China, Hengyang, China

**Keywords:** gastric cancer, pulmonary complications, prevention, best evidence, intervention strategies

## Abstract

**Background:**

Postoperative pulmonary complications in gastric cancer surgery significantly impact patient recovery and prognosis. These complications, including infections, can increase hospital stays and costs, and even lead to death. Numerous risk factors are involved, such as age, smoking history, and lung function. Although preventive measures exist, a unified and effective strategy is lacking. Therefore, researching and implementing effective prevention measures is crucial for improving patients’ postoperative quality of life and survival rates.

**Aim:**

To collate and summarize the best available evidence for the prevention of pulmonary complications in patients undergoing gastric cancer surgery, thereby providing a reference for the clinical development of relevant intervention strategies.

**Methods:**

A literature search was conducted in databases including BMJ Best Practice, UpToDate, JBI, Cochrane Library, PubMed, Embase, the Ontario Nurses Registration Network, the U.S. National Clinical Practice Guidelines, and MedLine, for documents related to the prevention of pulmonary complications in gastric cancer surgery patients. The search period extended from the inception of these databases to July 25, 2024. The quality of the literature was evaluated according to the standards of the Joanna Briggs Institute (JBI) Evidence-Based Health Care Center, and evidence was extracted from the included documents.

**Results:**

A total of 27 documents were ultimately included. The extracted content encompassed three areas: preoperative assessment, risk prevention and intervention measures, totaling 31 best evidences across five categories. The findings of our study underscore the significance of comprehensive preoperative assessments, such as the ARISCAT index for pulmonary risk evaluation, and stress the importance of preoperative interventions like inspiratory muscle training, smoking cessation, and oral care in mitigating postoperative pulmonary complications (PPCs) following gastric cancer surgery. We also advocate for the adoption of protective lung ventilation strategies during surgery and continuous pulse oximetry monitoring postoperatively, along with targeted treatments for specific complications.

**Conclusion:**

The best evidence extracted for the prevention of complications in gastric cancer surgery patients serves as a basis for evidence-based practice for the prevention of pulmonary complications in this patient group. Further research topics on pulmonary complications of gastric cancer, we recommend further optimization of preoperative assessment tools, investigation into the efficacy of smoking cessation programs, comparative studies on intraoperative ventilation strategies, development of postoperative rehabilitation programs, and research into culturally and resource-sensitive interventions to broaden the global applicability of these practices.

## 1 Introduction

Gastric cancer (GC) is one of the most common malignant tumors globally, ranking fifth among all cancers and third as a cause of cancer-related death (1). In 2018, it accounted for 5.7% of new cancer cases and 8.2% of cancer-related deaths (2). Comprehensive treatment centered around surgical resection continues to play a pivotal role in the management of gastric cancer (3). However, the incidence of postoperative complications after gastric cancer surgery ranges from 12.9% to 40.1% and is considered a significant factor affecting patient prognosis (3–5). Postoperative pulmonary complications (PPCs) are a common postoperative complication and the most frequent medium to long-term complication following major surgery, with an incidence rate of 1 to 23% (6, 7). Postoperative Pulmonary Complications (PPCs) refer to a variety of respiratory complications that occur after surgery. These complications primarily include, but are not limited to, pulmonary infections, respiratory failure, pleural effusions, atelectasis (lung collapse), pneumothorax, bronchospasm, aspiration pneumonia, pulmonary edema, and acute respiratory distress syndrome (ARDS). PPCs not only significantly impact patient recovery and mortality but also increase the demand for mechanical ventilation, intensive care, and prolonged hospital stays, thereby adding to the burden on healthcare services (8, 9). Therefore, the management of pulmonary complications has become one of the focal points in the perioperative management of gastric cancer and is paramount in perioperative nursing care.

Studies have indicated that postoperative pulmonary infections in gastric cancer patients can lead to recurrence and poor prognosis (10). Numerous risk factors contribute to the occurrence of pulmonary complications after gastric cancer surgery, with advanced age being a significant predictor of postoperative pneumonia and a leading cause of mortality, these risk factors also include smoking history, lung function, cardiovascular disease, diabetes, nutritional status, obesity, immunosuppression, and genetic factors, which can result in prolonged mechanical ventilation, extended hospital stays, increased mortality risk, poor prognosis, higher medical costs, and reduced quality of life. (11). Additionally, the surgical field for gastric cancer, particularly total gastrectomy, is close to the diaphragm, which can easily stimulate the diaphragm and lead to a reflexive decrease in its function, thereby affecting the recovery of postoperative pulmonary ventilation (12). Postoperative pain in gastric cancer patients can also result in poor spontaneous sputum expectoration, hindering the recovery of pulmonary function. Most gastric cancer patients suffer from malnutrition, and conditions such as anemia and hypoproteinemia can lead to pulmonary interstitial edema, affecting gas exchange and causing hypoxemia (13). The presence of a nasogastric tube postoperatively increases the incidence of pulmonary infection, as it can irritate the throat during swallowing, causing mucosal edema and airway compression, which impairs ventilation. Moreover, sputum can easily adhere to the tube’s walls during coughing, leading to bacterial colonization and an increased risk of pulmonary infection. The tube can also stimulate the throat and induce vomiting, raising the risk of aspiration (14, 15). Furthermore, factors such as prolonged surgery time, intraoperative bleeding, laparoscopic surgery, concurrent abdominal complications, and general anesthesia intubation all contribute to an increased incidence of pulmonary complications after gastric cancer surgery (13, 16).

With the advent of enhanced-recovery protocols, pulmonary rehabilitation during the perioperative period has gradually gained the attention of clinical researchers, reducing the occurrence of pulmonary complications after gastric cancer surgery to some extent. However, the current clinical practice lacks a systematic and standardized management process for perioperative pulmonary rehabilitation, and there is no universally recognized and effective intervention measures (13). Therefore, inconsistent guidance on the timing and duration of interventions, our study systematically synthesizes the best available evidence on postoperative complications in gastric cancer patients to provide patient-centered recommendations, address the optimal timing and duration of key interventions, and develop a comprehensive management strategy for postoperative pulmonary complications prevention in gastric cancer surgery patients, aiming to improve patient outcomes and reduce the healthcare burden.

## 2 Methods

This summary of best evidence was registered on Fudan University Evidence Based Nursing Center. The registered number is ES20245327.

### 2.1 Identification of the evidence-based issues

We used the PIPOST method as a guide to identify research questions (17), the evidence-based question was constructed as follows: Target Population (P): Patients in the perioperative period of gastric cancer surgery; Intervention (I): Preventive measures related to the occurrence of pulmonary infections; Practitioners (P): Clinical medical staff providing guidance for gastric cancer patients; Outcome (O): The incidence of postoperative pulmonary infections; Setting (S): Inpatient wards and specialty clinics; Type of Evidence (T): Best practice manuals, guidelines, expert consensuses, evidence summaries, recommended practices, and systematic reviews published in Chinese and English.

### 2.2 Search strategy

Chinese and English search terms were determined in accordance with the “6S” evidence pyramid model, searching from the most reliable sources downwards (18). Databases and websites including BMJ Best Practice, UpToDate, JBI, Cochrane Library, PubMed, Embase, Ontario Nurses Registration Network, U.S. National Clinical Practice Guidelines Database, and MedLine were searched.

Using the PIPOST approach, we developed a comprehensive search strategy for our research. The search utilized both subject words and free words. Subject words were” Stomach Neoplasms, Gastrointestinal Neoplasms, Digestive System Neoplasms, Pneumonia, Bacterial Healthcare-Associated Pneumonia, Guideline”. Free words were (“ Stomach Neoplasm*, Stomach tumor*, Stomach cancer*, Stomach carcinoma*, gastric cancer*, gastric Neoplasm*, gastric tumor*, gastric carcinoma*”) and (“pulmonary complications, pulmonary comorbidities, pulmonary infection, respiratory infection, lower respiratory infection, pneumonia, new pneumonia, new pulmonary infection, hospital-acquired pneumonia, aspiration pneumonia” and “guideline, Practice Guideline, Best Practice*, Recommendation*, Consensus*, Experts Opinion*, Systematic Reviews, Evidence Summaries, Best Practice Manuals”). This structured approach ensures a thorough exploration of relevant literature and guidelines related to stomach neoplasms and pneumonia.

A representative search strategy in Databases is presented in **Table 1**. The search period was from the inception of these databases to July 25, 2024.

**Table 1:** Search strategy in databases.

Search strategy in PubMed	
#1 stomach neoplasms[MeSH Terms]	
#2 ((((((Stomach Neoplasm*[Title/Abstract]) OR (Stomach tumor*[Title/Abstract]) OR (Stomach cancer*[Title/Abstract]) OR (Stomach carcinoma*[Title/Abstract]))) OR (gastric cancer*[Title/Abstract])) OR (gastric Neoplasm*[Title/Abstract])) OR (gastric tumor*[Title/Abstract])) OR (gastric carcinoma*[Title/Abstract])	
#3 #1AND#2	
#4 (((((((((pulmonary complications[Title/Abstract]) OR (pulmonary comorbidities[Title/Abstract])) OR (pulmonary infection[Title/Abstract])) OR (respiratory infection[Title/Abstract])) OR (lower respiratory infection[Title/Abstract])) OR (pneumonia[Title/Abstract])) OR (new pneumonia[Title/Abstract])) OR (new pulmonary infection[Title/Abstract])) OR (hospital-acquired pneumonia[Title/Abstract])) OR (aspiration pneumonia[Title/Abstract])	
#5 guideline[MeSH Terms]	
#6 (((((((((guideline[Publication Type]) OR (Practice Guideline[Publication Type])) OR (guideline*[Title/Abstract])) OR (Best Practice*[Title/Abstract])) OR (Recommendation*[Title/Abstract])) OR (Consensus*[Title/Abstract])) OR (Experts Opinion*[Title/Abstract])) OR (Systematic Reviews[Title/Abstract])) OR (Evidence Summaries[Title/Abstract])) OR (Best Practice Manuals[Title/Abstract])	
#7 #5AND#6	
#8 #3AND#4AND#7	
Search strategy in Cochrane Library	
#1 (Stomach Neoplasms):ti,ab,kw OR (Gastrointestinal Neoplasms):ti,ab,kw OR)(Digestive System Neoplasms):ti,ab,kw OR)(Neoplasm, Stomach):ti,ab,kw OR (Stomach Neoplasm):ti,ab,kw OR (Gastric Neoplasms):ti,ab,kw OR (Gastric Neoplasm):ti,ab,kw OR (Neoplasm, Gastric):ti,ab,kw OR (Neoplasms, Gastric):ti,ab,kw OR (Neoplasms, Stomach):ti,ab,kw OR (Cancer of Stomach):ti,ab,kw OR (Stomach Cancers):ti,ab,kw OR (Cancer of the Stomach):ti,ab,kw OR (Gastric Cancer):ti,ab,kw OR (Cancer*, Gastric):ti,ab,kw OR (Gastric Cancers):ti,ab,kw OR (Stomach Cancer):ti,ab,kw OR (Cancer*, Stomach):ti,ab,kw OR (Gastric Cancer, Familial Diffuse):ti,ab,kw OR (Stomach Neoplasms):ti,ab,kw OR (Gastrointestinal Neoplasm*):ti,ab,kw OR (Neoplasm*, Gastrointestinal):ti,ab,kw OR (Cancer of Gastrointestinal Tract):ti,ab,kw OR (Gastrointestinal Tract Cancer*):ti,ab,kw OR (Cancer*, Gastrointestinal):ti,ab,kw OR (Gastrointestinal Cancer*):ti,ab,kw OR (Cancer* of the Gastrointestinal Tract):ti,ab,kw OR (Digestive System Neoplasm*):ti,ab,kw OR (Neoplasm*, Digestive System):ti,ab,kw OR (Cancer* of Digestive System):ti,ab,kw OR (Digestive System Cancer*):ti,ab,kw OR (Cancer*, Digestive System):ti,ab,kw	
#2 (pneumonia):ti,ab,kw OR (Pneumonia, Bacterial):ti,ab,kw OR (Healthcare-Associated Pneumonia):ti,ab,kw OR (Pneumonia*):ti,ab,kw OR (Experimental Lung Inflammation*):ti,ab,kw OR (Inflammation, Experimental Lung):ti,ab,kw OR (Lung Inflammation*, Experimental):ti,ab,kw OR (Lobar Pneumonia*):ti,ab,kw OR (Pneumonia*, Lobar):ti,ab,kw OR (Pneumonitis*):ti,ab,kw OR (Lung Inflammation*):ti,ab,kw OR (Inflammation*, Lung):ti,ab,kw OR) OR (Inflammation*, Pulmonary):ti,ab,kw OR (Bacterial Pneumonia*):ti,ab,kw OR (Pneumonia*, Bacterial):ti,ab,kw OR (Healthcare Associated Pneumonia*):ti,ab,kw OR (Pneumonia*, Healthcare-Associated):ti,ab,kw OR (Nosocomial Pneumonia*):ti,ab,kw OR (Pneumonia*, Nosocomial):ti,ab,kw OR (Hospital Acquired Pneumonia*):ti,ab,kw OR (Pneumonia*, Hospital Acquired):ti,ab,kw OR (Healthcare-Associated Pneumonia*):ti,ab,kw	
#3 #1AND#2	
#4 (guideline):ti,ab,kw OR (Practice Guideline):ti,ab,kw OR (guideline*):ti,ab,kw OR (Best Practice*):ti,ab,kw OR (Experts Opinion*):ti,ab,kw OR (Systematic Reviews):ti,ab,kw OR (Recommendation*):ti,ab,kw OR (Consensus*):ti,ab,kw OR (Evidence Summaries):ti,ab,kw OR (Best Practice Manuals):ti,ab,kw	
#5 #3AND#4	
Search strategy in EMBASE	
#1 ‘Stomach Neoplasms’ OR ‘Gastrointestinal Neoplasms’ OR ‘Digestive System Neoplasms’ OR ‘Neoplasm, Stomach’ OR ‘Stomach Neoplasm’ OR ‘Gastric Neoplasms’ OR ‘Gastric Neoplasm’ OR ‘Neoplasm, Gastric’ OR ‘Neoplasms, Gastric’ OR ‘Neoplasms, Stomach’ OR ‘Cancer of Stomach’ OR ‘Stomach Cancers’ OR ‘Cancer of the Stomach’ OR ‘Gastric Cancer’ OR ‘Cancer*, Gastric’ OR ‘Gastric Cancers’ OR ‘Stomach Cancer’ OR ‘Cancer*, Stomach’ OR ‘Gastric Cancer, Familial Diffuse’ OR ‘Stomach Neoplasms’ OR ‘Gastrointestinal Neoplasm*’ OR ‘Neoplasm*, Gastrointestinal’ OR ‘Cancer of Gastrointestinal Tract’ OR ‘Gastrointestinal Tract Cancer*’ OR ‘Cancer*, Gastrointestinal’ OR ‘Gastrointestinal Cancer*’ OR ‘Cancer* of the Gastrointestinal Tract’ OR ‘Digestive System Neoplasm*’ OR ‘Neoplasm*, Digestive System’ OR ‘Cancer* of Digestive System’ OR ‘Digestive System Cancer*’ OR ‘Cancer*, Digestive System’	
#2 ‘pneumonia’ OR ‘bacterial pneumonia’ OR ‘health care associated pneumonia’ OR ‘Pneumonia*’ OR ‘Experimental Lung Inflammation*’ OR ‘Inflammation, Experimental Lung’ OR ‘Lung Inflammation*, Experimental’ OR ‘Lobar Pneumonia*’ OR ‘Pneumonia*, Lobar’ OR ‘Pneumonitis*’ OR ‘Lung Inflammation*’ OR ‘Inflammation*, Lung’ OR ‘Inflammation*, Pulmonary’ OR ‘Bacterial Pneumonia*’ OR ‘Pneumonia*, Bacterial’ OR ‘Healthcare Associated Pneumonia*’ OR ‘Pneumonia*, Healthcare-Associated’ OR ‘Nosocomial Pneumonia*’ OR ‘Pneumonia*, Nosocomial’ OR ‘Hospital Acquired Pneumonia*’ OR ‘Pneumonia*, Hospital Acquired’ OR ‘Healthcare-Associated Pneumonia*’	
#3 #1AND#2	
#4 ‘guideline’ OR ‘Practice Guideline’ OR ‘guideline*’ OR ‘Best Practice*’ OR ‘Experts Opinion’ OR ‘Systematic Reviews’ OR ‘Recommendation*’ OR	
‘Consensus*’ OR ‘Evidence Summaries’ OR ‘Best Practice Manuals’	
#5 #3AND#4	
Search strategy in JBI	
#1 (Stomach Neoplasms) or (Gastrointestinal Neoplasms) or (Digestive System Neoplasms) or (Neoplasm, Stomach) or (Stomach Neoplasm) or (Gastric Neoplasms) or (Gastric Neoplasm) or (Neoplasm, Gastric) or (Neoplasms, Gastric) or (Neoplasms, Stomach) or (Cancer of Stomach) or (Stomach Cancers) or (Cancer of the Stomach) or (Gastric Cancer) or (Cancer*, Gastric) or (Gastric Cancers) or (Stomach Cancer) or (Cancer*, Stomach) or (Gastric Cancer, Familial Diffuse) or (Stomach Neoplasms) or (Gastrointestinal Neoplasm*) or (Neoplasm*, Gastrointestinal) or (Cancer of Gastrointestinal Tract) or (Gastrointestinal Tract Cancer*) or (Cancer*, Gastrointestinal) or (Gastrointestinal Cancer*) or (Cancer* of the Gastrointestinal Tract) or (Digestive System Neoplasm*) or (Neoplasm*, Digestive System) or (Cancer* of Digestive System) or (Digestive System Cancer*) or (Cancer*, Digestive System)	
#2 (pneumonia) or (Pneumonia, Bacterial) or (Healthcare-Associated Pneumonia) or (Pneumonia*) or (Experimental Lung Inflammation*) or (Inflammation, Experimental Lung) or (Lung Inflammation*, Experimental) or (Lobar Pneumonia*) or (Pneumonia*, Lobar) or (Pneumonitis*) or (Lung Inflammation*) or (Inflammation*, Lung) or (Inflammation*, Pulmonary) or (Bacterial Pneumonia*) or (Pneumonia*, Bacterial) or (Healthcare Associated Pneumonia*) or (Pneumonia*, Healthcare-Associated) or (Nosocomial Pneumonia*) or (Pneumonia*, Nosocomial) or (Hospital Acquired Pneumonia*) or (Pneumonia*, Hospital Acquired) or (Healthcare-Associated Pneumonia*)	
#3 #1AND#2	
#4 (guideline) OR (Practice Guideline) OR (guideline*) OR (Best Practice*) OR (Experts Opinion) OR (Systematic Reviews) OR (Recommendation*) OR (Consensus*) OR (Evidence Summaries) OR (Best Practice Manuals)	
#5 #3AND#4	
Search strategy in WOS	
#1 TS=(“Stomach Neoplasms” OR “Gastrointestinal Neoplasms” OR “Digestive System Neoplasms” OR “Neoplasm, Stomach” OR “Stomach Neoplasm” OR “Gastric Neoplasms” OR “Gastric Neoplasm” OR “Neoplasm, Gastric” OR “Neoplasms, Gastric” OR “Neoplasms, Stomach” OR “Cancer of Stomach” OR “Stomach Cancers” OR “Cancer of the Stomach” OR “Gastric Cancer” OR “Cancer*, Gastric” OR “Gastric Cancers” OR “Stomach Cancer” OR “Cancer*, Stomach” OR “Gastric Cancer, Familial Diffuse” OR “Stomach Neoplasms” OR “Gastrointestinal Neoplasm*” OR “Neoplasm*, Gastrointestinal” OR “Cancer of Gastrointestinal Tract” OR “Gastrointestinal Tract Cancer*” OR “Cancer*, Gastrointestinal” OR “Gastrointestinal Cancer*” OR “Cancer* of the Gastrointestinal Tract” OR “Digestive System Neoplasm*” OR “Neoplasm*, Digestive System” OR “Cancer* of Digestive System” OR “Digestive System Cancer*” OR “Cancer*, Digestive System”)	
#2 TS=(“pneumonia” OR “Pneumonia, Bacterial” OR “Healthcare-Associated Pneumonia” OR “Pneumonia*” OR “Experimental Lung Inflammation*” OR “Inflammation, Experimental Lung” OR “Lung Inflammation*, Experimental” OR “Lobar Pneumonia*” OR “Pneumonia*, Lobar” OR “Pneumonitis*” OR “Lung Inflammation*” OR “Inflammation*, Lung” OR “Inflammation*, Pulmonary” OR “Bacterial Pneumonia*” OR “Pneumonia*, Bacterial” OR “Healthcare Associated Pneumonia*” OR “Pneumonia*, Healthcare-Associated” OR “Nosocomial Pneumonia*” OR “Pneumonia*, Nosocomial” OR “Hospital Acquired Pneumonia*” OR “Pneumonia*, Hospital Acquired” OR “Healthcare-Associated Pneumonia*”)	
#3 #1AND#2	
#4 TS=(“guideline” OR “Practice Guideline” OR “Best Practice*” OR “Experts Opinion” OR “Systematic Reviews” OR “Recommendation*” OR “Consensus*” OR “Evidence Summaries” OR “Best Practice Manuals”)	
#5 #3AND#4	

### 2.3 Inclusion and exclusion criteria

To ensure our study includes the most relevant and high-quality evidence, we have established detailed inclusion criteria that focus on patients of all ages undergoing gastric cancer surgery, prioritize literature on preventive measures for postoperative pulmonary complications, consider only Chinese and English documents, emphasize high-quality study designs, limit our search to publications from the inception of databases until July 25, 2024, exclude abstracts without full texts, and prioritize literature with clear clinical applicability to provide scientific guidance for preventing pulmonary complications after gastric cancer surgery.

### 2.4 Quality assessment

The quality assessment of guidelines was conducted using the updated Clinical Guidelines Research and Evaluation System II (AGREE II) from 2012 (19); expert consensus was evaluated using the quality assessment tool for expert opinions and professional consensus articles from the Australian JBI Evidence-Based Practice Center (2016); systematic reviews were assessed using the systematic review tool from the Australian JBI Evidence-Based Practice Center (2016); the quality assessment of clinical decisions, best practices, and evidence summaries traced back to the original literature they were based on, with appropriate evaluation standards selected based on the type of literature.

### 2.5 Data extraction

**Figure 1:**
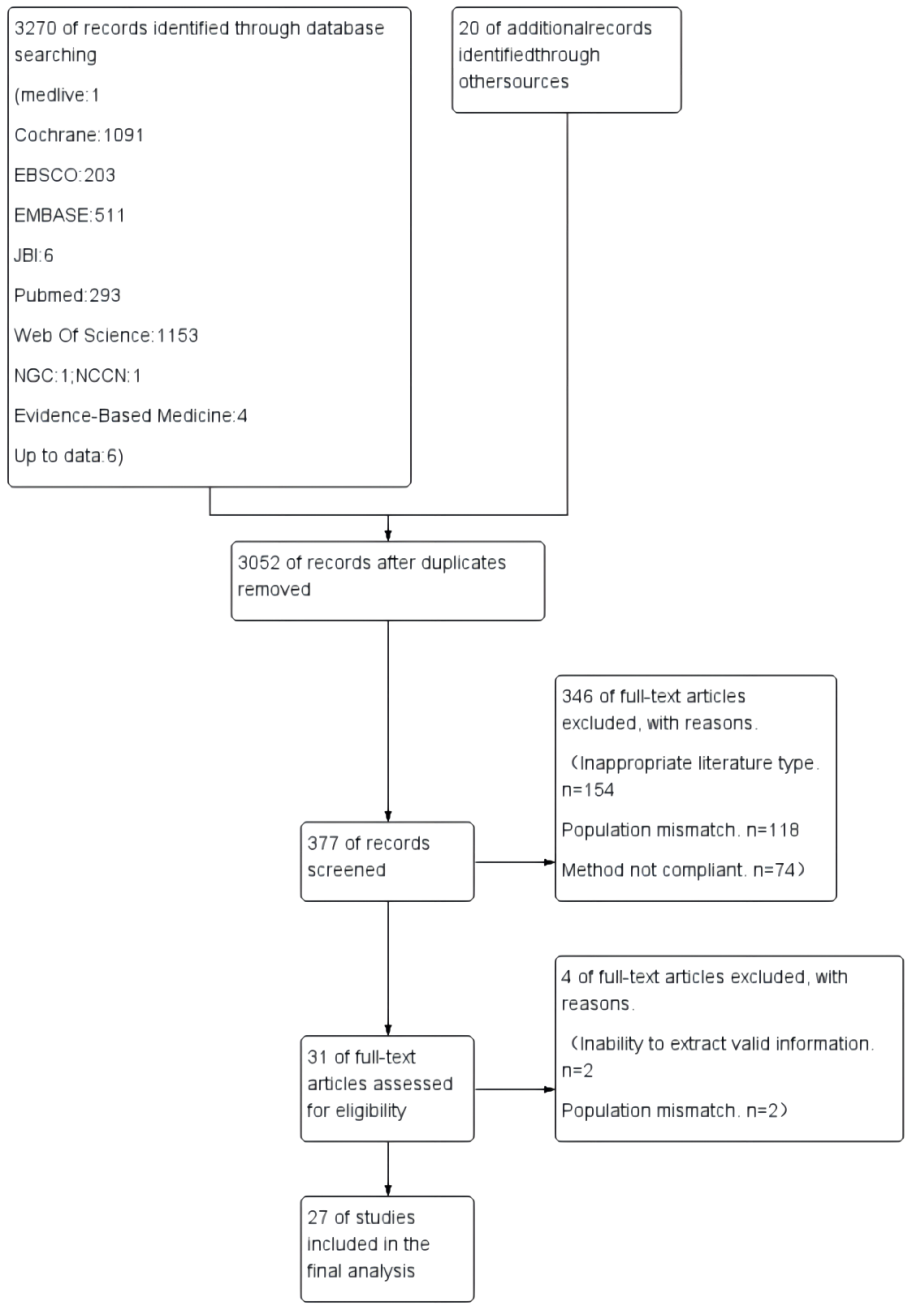
Data extraction.

Two researchers trained in systematic review methodology independently searched, screened, and extracted data, cross-checking after the initial search. The preliminary search results were imported into EndNote X9 for deduplication, followed by full-text screening based on the titles and abstracts, and finally, the full texts were rescreened to include the final set of articles. Disagreements were resolved through consensus by a third researcher. A total of 27 articles were included in the study (see **Figure 1**).

### 2.6 Evidence description and summary

Two researchers independently read each included article, extracted evidence content, and organized and summarized the evidence by theme. The study prioritized evidence-based evidence, high-quality evidence, and the most recently published authoritative literature. The evidence was graded using the Australian JBI Evidence-Based Nursing Center’s pre-grading system and evidence recommendation level system (2014 edition), in conjunction with an expert argumentation meeting that considered the “FAME” framework (feasibility, appropriateness, meaningfulness, and effectiveness) and the JBI evidence recommendation level principle. In this framework, Level A represents a strong recommendation, while Level B indicates a weak recommendation. The expert argumentation meeting involved the participation of 10 experts, including 5 gastrointestinal surgeons, 3 oncology nurses, and 2 anesthesiologists. All 10 experts possess senior professional titles and hold a master’s degree or higher. Evidence was categorized into levels 1 to 5, with level 1 being the highest and level 5 the lowest, based on the type of research design and the consensus reached by the expert panel. Three members of the research team, trained in systematic review methodology and evidence-based nursing, have extensive experience in evidence extraction and grading, and were part of the expert argumentation process.

## 3 Results

### 3.1 Characteristics of the included evidence

In this study, the literature screening process began with an initial database search, identifying 3270 records, and an additional 20 records were identified through other sources, totaling 3291 records. After deduplication, 3052 records remained. Following the acquisition of full texts, 346 were excluded due to inappropriate literature types (154), population mismatch (118), or non-compliant methods (74). Subsequently, 377 records were further screened. During the full-text assessment phase, an additional 4 were excluded due to the inability to extract valid information (2) or population mismatch (2). Ultimately, 27 studies were included in the final analysis. This flowchart clearly illustrates how the research team rigorously sifted through a large number of initial literature to select high-quality documents that meet the research criteria, ensuring the reliability and validity of the study’s results. The literature screening flowchart is shown in **Figure 1**. The general characteristics of the included literature are shown in **Table 2**.

**Table 2:** General characteristics of included literature (n=27).

Author(s)	Publication Year	Type	Source	Topic
Joyce et al. (20).	2023	Clinical Decision	UpToDate	Prehabilitation in Surgical Patients
Smetana (21).	2024	Clinical Decision	UpToDate	Measures to Reduce Postoperative Pulmonary Complications in Adults
Smetana (22).	2024	Clinical Decision	UpToDate	Preoperative Pulmonary Risk Assessment
Conde (23)	2024	Clinical Decision	UpToDate	Treatment of Various Airway and Pulmonary Complications in Adults After Surgery
Hert et al. (24).	2018	Guidelines	UpToDate	Preoperative Assessment for Elective Noncardiac Surgery in Adults
Chinese Medical Association of Surgery (25).	2021	Guidelines	CNKI	Clinical Practice of Enhanced Recovery After Surgery in China
The American Society of Anesthesiologists (Updated 2023) (26).	2023	Guidelines	UpToDate	Preoperative Fasting and Medications to Reduce the Risk of Pulmonary Aspiration
Zaccagnini (27).	2021	Evidence Summary	JBI	Postoperative Respiratory Depression (Prevention): Pulse Oximetry and Carbon Dioxide Monitoring
Overall (28).	2024	Evidence Summary	JBI	Preoperative Strategies for Postoperative Pulmonary Complications
Overall and Mevin (29).	2024	Evidence Summary	JBI	Intraoperative Strategies for Postoperative Pulmonary Complications
Overall (30).	2024	Evidence Summary	JBI	Postoperative Strategies for Postoperative Pulmonary Complications
Pamaiahgari (31).	2022	Evidence Summary	JBI	Preoperative Smoking Cessation Intervention
Myers et al. (32).	2011	Systematic Review	PubMed	Preoperative Smoking Cessation and Postoperative Complications
Pedersen et al. (33).	2014	Systematic Review	Cochrane Library	Perioperative Monitoring of Pulse Oximetry
Thomsen et al. (34).	2014	Systematic Review	Cochrane Library	Interventions for Preoperative Smoking Cessation
Katsura et al. (35).	2015	Systematic Review	Cochrane Library	Effects of Preoperative Inspiratory Muscle Training on Postoperative Pulmonary Complications in Adults Undergoing Cardiac and Abdominal Surgery
Lam et al. (36).	2017	Systematic Review	PubMed	Continuous Pulse Oximetry and Capnography Monitoring for Postoperative Respiratory Depression and Adverse Events
Wang et al. (37).	2018	Systematic Review	PubMed	Meta-Analysis of ERAS Application in Gastric Cancer Patients
Hughes et al. (38).	2019	Systematic Review	PubMed	Preoperative Training Before Major Abdominal Surgery
Lau et al. (39).	2020	Systematic Review	PubMed	Perioperative Rehabilitation Training for Patients with Gastrointestinal Tumors
Odor et al. (40).	2020	Systematic Review	PubMed	Perioperative Management to Prevent Postoperative Pulmonary Complications
Assouline et al. (41).	2021	Systematic Review	PubMed	Preoperative Exercise Training to Prevent Postoperative Pulmonary Complications in Adults Undergoing Major Surgery
Mortensen et al. (42).	2014	Consensus	PubMed	Perioperative Nursing for Gastric Resection
Rochester et al. (43).	2015	Consensus	PubMed	Implementation, Use, and Adoption of Enhanced Recovery After Surgery
Carli et al. (44).	2017	Consensus	PubMed	Surgical Rehabilitation for Cancer Patients
Gelb et al. (45).	2018	Consensus	Cochrane Library	International Standards for Safe Anesthesia Practice
Tew et al. (46).	2018	Consensus	PubMed	Preoperative Exercise Training in Patients Undergoing Major Noncardiac Surgery

### 3.2 Literature quality evaluation results

#### 3.2.1 Quality assessment results of guidelines

The quality of the guidelines was assessed using the 2012 updated Clinical Guidelines Research and Evaluation System II (AGREE II). A total of three guidelines were included in this study. The Intraclass Correlation Coefficient (ICC) values for the assessment results from four evaluators were all greater than 0.75, indicating a high level of consistency among the evaluators. The recommendation levels were as follows: A grade (directly recommended) for areas scoring 60% or above; B grade (requires modification and improvement) for areas with scores below 60% but with at least three areas scoring 30% or above; and C grade (not recommended for the time being) for areas with at least three scores below 30%. Overall, the quality of the three guidelines was high, with detailed standardized scores and recommendation levels presented in **Table 3**.

**Table 3:** Standardized percentages of guideline dimensions and evaluation results (n=3).

Author(s)	Scope and Purpose	Participants	Rigor of Guideline Development	Clarity of Presentation	Applicability	Editor Independence	≥60% Domains Count	≥30% Domains	ICC	Recommendation Level
Hert et al. (45).	96	72	85	97	89	88	6	6	0.862	A
The American Society of Anesthesiologists (26).	99	90	80	92	70	100	6	6	0.892	A
Chinese Medical Association of Surgery (26).	93	64	51	70	76	71	5	6	0.894	B

The “≥60% Domains Count” column indicates the number of domains scoring 60% or above. The “≥30% Domains Count” column indicates the number of domains scoring 30% or above. “ICC” stands for Intraclass Correlation Coefficient, a measure of reliability. The “Recommendation Level” column provides the overall recommendation level of the guidelines.

#### 3.2.2 Quality assessment results of expert consensus

The quality of expert consensus documents was evaluated using the quality assessment tool for expert opinions and professional consensus articles from the Australian JBI Evidence-Based Practice Center (2016). A total of five expert consensus documents were included in this study, with specific evaluation results shown in **Table 4**.

**Table 4:** Quality evaluation results of expert consensus literature (n=5).

Items	Carli et al (45)	Rochester et al (43)	Gelb et al (45)	Tew et al (46)	Mortensen et al (42)
1. Is the source of the opinion clearly identified?	Y	Y	Y	Y	Y
2. Does the source of opinion have standing in the field of expertise?	Y	Y	Y	Y	Y
3. Are the interests of the relevant population the central focus of the opinion?	Y	Y	Y	Y	Y
4. Is the stated position the result of an analytical process, and is there logic in the opinion expressed?	Y	Y	Y	Y	Y
5. Is there reference to the extant literature?	Y	Y	Y	Y	Y
6. Is any incongruence with the literature/sources logically defended?	Y	Y	Y	Y	Y

Y, Yes; N, No; CA, Unclear; NA, Not Applicable.

#### 3.2.3 Quality assessment results of systematic review

The quality of systematic reviews was assessed using the systematic review tool from the Australian JBI Evidence-Based Practice Center (2016). A total of ten systematic reviews were included in this study, with specific evaluation results shown in **Table 5**.

Table 5Results of systematic review literature quality assessment (n=10).

**Table:** 

Items	Katsura et al (35)	Thomsen et al (34)	Myers et al (32).	Assouline et al (41).	Hughes et al (38).
1. Whether the evidence- based questions raised are clear and unambiguous?	Y	Y	Y	Y	Y
2. Whether the literature inclusion criteria were appropriate for this evidence- based question?	Y	Y	Y	Y	Y
3. Is the search strategy appropriate?	Y	Y	CA	Y	CA
4. The adequacy of databases or resources for searching literature?	Y	Y	Y	Y	Y
5. Whether the literature quality evaluation criteria used are appropriate?	Y	Y	Y	Y	Y
6.Whether two or more reviewers independently complete the quality evaluation of the literature?	Y	Y	Y	Y	Y
7. Whether to take certain measures to reduce errors when extracting data?	Y	CA	CA	CA	CA
8. Whether the methods of pooling studies were appropriate?	Y	Y	Y	Y	Y
9. Whether the possibility of publication bias was assessed?	Y	CA	CA	Y	Y
10. Whether the proposed policy or practice recommendations are based on the results of systematic reviews?	Y	Y	Y	Y	Y
11. Whether the proposed directions for further research are appropriate?	Y	Y	Y	Y	Y

Y, Yes; N, No; CA, Unclear; NA, Not Applicable.

**Table:** 

Items	Lam et al (36).	Pedersen et al (33).	Odor et al (40).	Lau et al (39).	Wang et al (37).
1. Whether the evidence- based questions raised are clear and unambiguous?	Y	Y	Y	Y	Y
2. Whether the literature inclusion criteria were appropriate for this evidence- based question?	Y	Y	Y	Y	Y
3. Is the search strategy appropriate?	Y	Y	Y	CA	Y
4. The adequacy of databases or resources for searching literature?	Y	Y	Y	Y	Y
5. Whether the literature quality evaluation criteria used are appropriate?	Y	Y	Y	Y	Y
6.Whether two or more reviewers independently complete the quality evaluation of the literature?	Y	Y	Y	CA	Y
7. Whether to take certain measures to reduce errors when extracting data?	CA	Y	Y	CA	Y
8. Whether the methods of pooling studies were appropriate?	Y	CA	Y	Y	Y
9. Whether the possibility of publication bias was assessed?	Y	Y	Y	Y	Y
10. Whether the proposed policy or practice recommendations are based on the results of systematic reviews?	Y	Y	Y	Y	Y
11. Whether the proposed directions for further research are appropriate?	Y	Y	Y	Y	Y

Y, Yes; N, No; CA, Unclear; NA, Not Applicable.

#### 3.2.4 Quality assessment results of clinical decision and evidence summary

Clinical decisions and evidence summaries were included based on the extraction of original literature from high-quality systematic reviews or randomized controlled trials. A total of four clinical decisions and five evidence summaries were included in this study.

All literature was independently assessed by two researchers trained in standardized evidence-based methodology. In cases of disagreement, a decision was made in consultation with a third teacher experienced in evidence-based methodology and clinical practice.

### 3.3 Evidence organization and grading

Evidence was extracted, evaluated, and organized to generate 31 pieces of evidence for the prevention of postoperative pulmonary complications following gastric cancer surgery. This comprehensive set includes preoperative assessment, risk prevention measures and intervention strategies. These areas are categorized into five distinct groups.

Two researchers, trained in systematic evidence review methods, utilized the 2014 version of the Joanna Briggs Institute (JBI) evidence pre-grading system to classify the included evidence. The evidence was graded on a scale of 1 to 5, with 1 representing the highest level of evidence and 5 the lowest. For detailed grading,refer to **Table 6**.

**Table 6:** Best evidence summary for preventing postoperative pulmonary complications after gastric cancer surgery.

Subject of Evidence		Evidence Content	Evidence Level	Recommendation
Risk Assessment	Preoperative Assessment	1. Comprehensive medical history and physical examination are crucial for assessing the risk of postoperative pulmonary complications. (22, 25, 26)	Level 4	B
		2. Sufficient time should be allowed for preoperative assessment to implement any desirable preoperative interventions that may improve outcomes. (22)	Level 2	C
		3. Functional measurements, such as the degree of independence, frailty, and anxiety, should be implemented in the preoperative assessment. (25)	Level 1	B
		4. While a nurse or another physician can perform the preoperative assessment, it is recommended to be done by an anesthesiologist. (24, 45)	Level 1	C
		5. In specific patients, pulmonary function tests (PFTs), chest X-rays, and exercise tolerance tests may identify risk factors that need attention before surgery. (22, 24)	Level 2	B
		6. For patients over 50 undergoing high-risk surgery, or where clinical assessment suggests cardiac or pulmonary disease, chest X-ray results from within the last 6 months are recommended. (22, 24)	Level 3	C
		7. The ARISCAT index is recommended for assessing pulmonary risk before major non-cardiac surgery. (22, 24)	Level 2	A
		8. Patients with long-term diabetes should undergo a careful airway assessment. (24)	Level 2	C
		9. The preoperative assessment for obese patients should at least include the STOP-BANG questionnaire, clinical evaluation, ECG, oximetry, or polysomnography. (24)	Level 2	B
		10. Diagnostic pulmonary function tests are not recommended to assess the risk of postoperative complications in non-cardiac and non-thoracic surgery patients. (22, 24)	Level 1	C
		11. Computer-based preoperative assessment tools are recommended whenever possible; they are based on well-designed, standardized questionnaires and can improve the quality of assessment. (22)	Level 2	B
Risk Prevention	Inspiratory muscle training	12. Patients scheduled for abdominal, thoracic, or cardiac surgery should implement a multifaceted preoperative risk reduction strategy to prevent postoperative pulmonary complications, including physical activity such as aerobic exercise and respiratory muscle training. (20, 28, 30, 39, 43, 44)	Level 1	A
		13. Preoperative inspiratory muscle training is recommended to reduce the incidence of postoperative atelectasis, pneumonia, and hospital stay. (35, 37, 41)	Level 2	A
		14. Inspiratory muscle training should be conducted by a physiotherapist or physician. (28)	Level 1	A
		15. Preoperative inspiratory muscle training, such as lung expansion exercises and voluntary deep breathing, is recommended for patients at risk of developing pulmonary complications, including coughing and incentive spirometry. (40)	Level 1	A
		16. Inspiratory muscle training should begin at least two weeks before surgery, with five to seven sessions per week, each lasting 15-30 minutes. (28)	Level 1	A
	Quit smoking	17. Preoperative smoking cessation is recommended to encourage long-term abstinence and reduce postoperative wound healing complications. (31)	Level 1	A
		18. A multifaceted program for smoking cessation, including nicotine replacement therapy, should be considered to assist patients in quitting before elective surgery. (31)	Level 1	A
		19. Patients undergoing elective surgery should be advised to quit smoking as soon as possible, preferably at least eight weeks before surgery. (28, 31, 42)	Level 2	C
		20. There is insufficient evidence to suggest that short-term smoking cessation (<4 weeks) reduces the incidence of postoperative complications. (32, 34)	Level 1	A
	Oral care	21. Preoperative oral care with chlorhexidine mouthwash is recommended and can effectively reduce the incidence of postoperative pulmonary complications, although the optimal volume and frequency of use are not well-defined and should be determined by the clinician. (21, 28)	Level 1	B
	Intraoperative Prevention	22. Protective lung ventilation is recommended for adult patients undergoing surgery, including positive end-expiratory pressure (PEEP) ≤5cmH2O, tidal volume (TV) ≤8ml/kg, and intermittent alveolar recruitment maneuvers (AMR). (21, 29)	Level 1	B
Intervention Measures	Observation	23. Pulse oximetry is recommended for continuous monitoring of oxygen saturation levels in the blood and should be worn on the finger whenever possible. (27, 33, 36)	Level 1	B
		24. Pulse oximetry readings should not be the sole tool for assessing a patient’s clinical condition. (27, 33, 36)	Level 2	B
	Complication Management	25. If patients develop hypoxemia and/or increased respiratory effort due to postoperative atelectasis without significant respiratory secretions, continuous positive airway pressure (CPAP) is recommended. (21, 30)	Level 2	C
		26. For patients with significant respiratory secretions and clinically important postoperative atelectasis, the use of mucolytic agents and frequent suctioning and chest physiotherapy (i.e., positioning and percussion) are recommended. (21)	Level 2	C
		27. Bronchoscopic intervention should not be routinely performed before suctioning and chest physiotherapy; it should be reserved for patients where these measures are ineffective. (23)	Level 2	B
		28. Short-acting inhaled beta-2 agonists, such as salbutamol or oxitropium, are recommended as first-line treatment for patients with postoperative bronchospasm. (23)	Level 1	A
		29. Pneumonia often occurs within 5 days postoperatively. Treatment includes obtaining respiratory tract specimens for microbiological analysis, and once microbiological data are available and the effectiveness of empirical treatment has been assessed, an antimicrobial regimen should be tailored to the patient’s condition. (23)	Level 2	B
		30. Acute upper airway obstruction usually occurs immediately after surgery and requires immediate assessment by a physician capable of performing tracheal intubation. (23)	Level 2	B
		31. A small amount of pleural effusion may occur after abdominal surgery, most of which resolve on their own within a few days and do not require intervention. If signs of infection occur (fever, chest infiltration, increased productive cough), diagnostic assessment of the pleural effusion is necessary. (23)	Level 3	A

## 4 Discussion

### 4.1 Current status of evidence for the prevention of postoperative pulmonary complications and the value of utilizing related evidence

The National Surgical Quality Improvement Program (NSQIP) reported a 6% incidence of postoperative pulmonary complications (PPCs) in 165,196 patients who underwent major abdominal surgery, with these patients facing higher mortality, ICU admission rates, and longer hospital stays. An additional NSQIP study revealed a 10.25% mortality rate within 30 days post-surgery among outpatients with PPCs. Postoperative lung volume reduction, particularly after thoracic and upper abdominal surgeries, increases PPC risk by causing restrictive lung volume decrease, reduced tidal volume, loss of sighing respiration, and elevated respiratory rates. Gastric cancer surgery, especially total gastrectomy, is prone to irritate the diaphragm, affecting pulmonary ventilation recovery.

In China, there’s a recognized need for improved emphasis on preventing PPCs in gastric cancer surgery, with current practices lacking standardized management for risk assessment and intervention. This study compiles evidence to aid medical staff in managing PPCs post-gastric cancer surgery, aiming to early identify high-risk patients and implement preventive measures to enhance medical care quality. It includes relevant strategies like preoperative aerobic training and respiratory exercises, essential for respiratory muscle preparation in gastric cancer patients, to be tailored to clinical practice.

### 4.2 Risk assessment of postoperative pulmonary complications after gastric cancer surgery

Postoperative pulmonary complications (PPCs) are a significant concern following gastric cancer surgery, necessitating a standard risk assessment as part of preoperative evaluations. The ARISCAT score, developed by Canet J et al., is a recommended tool for this purpose, incorporating seven risk factors: age, recent respiratory infection, preoperative SpO2, anemia, surgical site, duration, and emergency status. It stratifies patients into low (≤26 points), medium (27-44 points), and high (≥45 points) risk categories. Wang Xiaomei’s external validation confirmed its efficacy in major abdominal surgeries.

To enhance the accuracy of risk assessment and personalized preoperative preparation, it is advisable for anesthesiologists, given their expertise in anesthesia management, to conduct these evaluations. The ARISCAT score’s inclusion of both preoperative and intraoperative variables facilitates timely postoperative assessments and interventions. Interdisciplinary collaboration, involving medical staff and anesthesiologists, is essential for a comprehensive approach to pulmonary complication prevention, ensuring that patients receive tailored recommendations that address their overall health needs.

### 4.3 Risk prevention of postoperative pulmonary complications after gastric cancer surgery

Evidence 12 to 20 highlights preventive strategies for gastric cancer surgery patients, with a focus on inspiratory muscle training (Evidence 12-16). This training, guided by professionals like physiotherapists, enhances respiratory muscle strength, exercise capacity, and alleviates dyspnea, starting at least two weeks pre-surgery with 5-7 sessions weekly. The optimal timing for intervention is tailored to individual patient circumstances. Preoperative smoking cessation (Evidence 17-18) is crucial, with long-term cessation recommended to reduce postoperative complications, though the effectiveness of short-term cessation is debated. Patients are advised to quit at least eight weeks before elective surgery to mitigate pulmonary risks. Preoperative oral care, particularly with chlorhexidine mouthwash, is emphasized to reduce oral pathogens and the risk of aspiration pneumonia. The frequency and volume of mouthwash use should be determined by oral hygiene status and clinical judgment. Intraoperatively, protective lung ventilation is advised for adult patients, including PEEP ≤5cmH2O, TV ≤8ml/kg, and AMR, to prevent alveolar overexpansion and atelectasis. The development and adjustment of personalized ventilation strategies are essential for patient safety and recovery.

In summary, a comprehensive preoperative regimen that includes inspiratory muscle training, smoking cessation, oral hygiene, and protective lung ventilation is vital for mitigating pulmonary complications in gastric cancer surgery, emphasizing the need for personalized care to optimize patient outcomes.

### 4.4 Management of postoperative pulmonary complications after gastric cancer surgery

Evidence 21 to 31 emphasizes critical intervention measures for preventing postoperative pulmonary complications (PPCs) after gastric cancer surgery. Key strategies include continuous monitoring with pulse oximeters to detect oxygen saturation levels below 95%, prompting immediate medical intervention and comprehensive patient assessment. Early detection and management of PPCs are essential for patient recovery.

For managing specific complications, physical therapy, such as sputum suction and chest percussion, is recommended for patients with respiratory tract secretions or atelectasis. Postoperative bronchospasm is treated with short-acting inhaled beta-2 agonists like salbutamol or oxitropium. Pneumonia treatment should be guided by microbiological analysis to ensure rational and targeted antibiotic use.

In cases of acute upper airway obstruction post-surgery, prompt assessment by a doctor skilled in tracheal intubation is crucial. While minor postoperative pleural effusions may resolve without intervention, signs of infection necessitate diagnostic assessment. These measures underscore the importance of proactive and tailored approaches to managing PPCs for optimal patient outcomes.

### 4.5 Comparison of evidence and resolving conflicting viewpoints

We acknowledge the importance of comparing and contrasting different evidence sources, particularly when there are conflicting viewpoints. Such an analysis is crucial for understanding how these differences may impact clinical practice, especially in the context of gastric cancer surgery. For example, while some guidelines advocate for aggressive preoperative pulmonary rehabilitation, others prioritize optimizing nutritional status before surgery. Similarly, there are differing opinions on the optimal duration of smoking cessation before surgery, with some sources suggesting a longer duration for better outcomes, while others emphasize the immediate need for surgery due to the aggressive nature of gastric cancer. The differences in these recommendations can lead to variations in clinical practice. For instance, the approach to preoperative preparation may differ significantly between institutions, potentially affecting patient outcomes. To reconcile these differences, we have taken the following steps. Firstly, we have contextualized each recommendation within the specific patient population and surgical context it was derived from, highlighting the need for tailored approaches based on individual patient needs. Secondly, we have synthesized the evidence to identify common themes and best practices that can be universally applied, while also acknowledging the areas where further research is needed to resolve discrepancies. Last but not least, we emphasize the importance of clinical judgment in interpreting these guidelines, advocating for a flexible approach that considers the unique circumstances of each patient.

### 4.6 Applicability and limitations of evidence

Gastric cancer patients may experience postoperative recovery and complications differently due to factors such as malnutrition and the impact of surgery on gastrointestinal function. Therefore, the applicability of the evidence must consider these unique aspects of gastric cancer treatment and recovery. Given the specific challenges faced by gastric cancer patients, it is crucial to tailor intervention measures to this patient population. This includes considerations for preoperative optimization of nutritional status, postoperative pain management protocols that facilitate pulmonary toilet, and targeted pulmonary rehabilitation programs. The evidence must be critically assessed and adapted to the specific needs of gastric cancer surgery patients. This involves considering local epidemiological data, resource constraints, and cultural sensitivities to ensure that the interventions are both relevant and effective.

The evidence we synthesized spans various surgical procedures, including abdominal, thoracic, and cardiac surgeries. While this broad perspective is valuable, it also means that the evidence may not fully capture the unique aspects of gastric cancer surgery, such as the specific impacts on pulmonary function due to the proximity of the surgery to the diaphragm. The evidence is derived from multiple regions and cultures, which may influence the practices and outcomes. These differences can affect the generalizability of the findings to gastric cancer surgery patients in different settings. The implementation of certain interventions may be limited by the availability of resources, which can vary significantly between healthcare systems. This variability could affect the feasibility of applying the evidence in all clinical environments.

## 5 Conclusion

This study summarizes the best evidence for the prevention of postoperative pulmonary complications in patients after gastric cancer surgery. The evidence comes from both domestic and international systematic evaluations, guidelines, and authoritative expert consensuses, and is of a high level with strong credibility. It can provide a basis for decision-making and clinical nursing practice by medical staff in relevant departments. However, the evidence included in this study comes from various countries, and there are differences in culture, environment, and resources in hospitals from different countries. It is recommended that relevant departments, in conjunction with the specific circumstances of their unit and patients, fully consider the applicability and feasibility of the evidence to develop personalized management plans for the prevention of postoperative pulmonary complications in patients after gastric cancer surgery, ensuring patient safety.
